# Outpatient assessment of bronchopulmonary dysplasia using point of care lung ultrasound

**DOI:** 10.3389/fped.2026.1733693

**Published:** 2026-04-02

**Authors:** Nicole Stephenson, William Stoudemire, Daniel Park, Lang Li, Charles Esther, Ria Dancel, Benjamin Smith, Feng-Chang Lin, Stephanie D. Davis

**Affiliations:** 1 Department of Pediatrics, The University of North Carolina School of Medicine, Chapel Hill, NC, United States; 2 Department of Biostatistics, Gillings School of Global Public Health, The University of North Carolina at Chapel Hill, Chapel Hill, NC, United States; 3 Department of Medicine, The University of North Carolina School of Medicine, Chapel Hill, NC, United States; 4 Department of Radiology, The University of North Carolina School of Medicine, Chapel Hill, NC, United States

**Keywords:** bronchopulmonary dysplasia, lung point-of-care ultrasound, lung ultrasound, lung ultrasound scores, respiratory morbidity

## Abstract

**Background:**

Lung point-of-care ultrasound (POCUS) is an emerging technology for assessment of neonatal lung diseases. Prior studies have described lung ultrasound scores (LUS) in bronchopulmonary dysplasia (BPD). However, few have assessed LUS in patients with BPD beyond the initial neonatal intensive care unit (NICU) hospitalization.

**Methods:**

We performed a cross-sectional study to evaluate outpatient LUS abnormalities and respiratory morbidity in children with and without a history of BPD. We hypothesized that infants with severe BPD would have higher (more abnormal) LUS compared to healthy infants. Eligible children received a single lung ultrasound evaluation during an outpatient clinic visit. The lung ultrasound images were analyzed and scored (0–18). A LUS greater than 0 was considered abnormal.

**Results:**

125 participants aged 0–24 months were enrolled, including 26 healthy full-term children (controls) and 45 preterm children without BPD (Grade 0). 16, 26, and 12 were enrolled with Grade 1, 2 and 3 BPD, respectively. Lung ultrasound scores were significantly higher in children with Grade 2 and Grade 3 BPD (mean LUS of 0.68 and 2.67, respectively), compared to healthy term children (mean LUS of 0.11). However, 91 preterm participants, including some with severe BPD, had normal ultrasounds (LUS = 0). Adding abnormal LUS to a model including established clinical risk factors significantly improved the model's ability to identify which children with BPD were likely to have a history of hospital readmissions (AUC increased from 0.718 to 0.785). However, due to low inter-observer agreement, these findings should be interpreted as exploratory and observer-dependent.

**Conclusion:**

Lung POCUS may be a feasible adjunctive assessment tool for children with BPD, and abnormal LUS may be associated with respiratory-related hospital readmissions. However, normal LUS findings were common, including among some children with severe BPD. Longitudinal studies with a larger cohort are needed to further evaluate its use and limitations.

## Introduction

Bronchopulmonary dysplasia (BPD), a chronic respiratory condition associated with prematurity, is caused by injury and impaired development of the premature lung. In the United States, BPD is the most common chronic lung disease in infancy, impacting 10,000–15,000 infants annually, including 40%–50% of infants born less than 28 weeks gestational age (1). Importantly, BPD is the most common complication and morbidity of prematurity (2). Despite advances in neonatal care, the incidence of BPD has increased due to improved survival rates of extremely low gestational age neonates (2). For infants who survive, BPD is associated with longer hospitalization lengths, an increased risk of respiratory and cardiovascular comorbidities, neurodevelopmental delays, impaired growth, and an increased risk of hospital readmissions. Though a diagnosis of BPD is defined based on the amount of respiratory support needed at 36 weeks corrected for gestational age (CGA) (3), imaging is often used to evaluate respiratory complications associated with BPD (e.g., cystic lesions, pneumothorax, and pulmonary edema), as well as for reassessment of clinical disease. Common imaging studies for evaluating lung disease secondary to BPD include chest radiography, computed tomography of the chest (CT chest), and occasionally MRI (magnetic resonance imaging) of the chest (4). However, these imaging techniques have disadvantages including cost, lack of availability, and exposure to radiation, limiting their role in management of BPD.

Point-of-care ultrasound (POCUS) has emerged as a tool to evaluate lung abnormalities due to its advantages including easy availability, portability, real-time results, and lack of radiation. Defining the utility of lung ultrasound in BPD may help to reduce cumulative radiation associated with recurrent x-rays in childhood. The benefit of lung ultrasound in identifying abnormalities in other chronic lung diseases, such as in asthma and chronic obstructive pulmonary disease (COPD), is well described (5, 6). Recent literature has reported the diagnostic value of lung ultrasound findings in preterm infants with respiratory distress syndrome and demonstrated the ability of lung ultrasound to predict development of BPD (7–10). Furthermore, the lung ultrasound score (LUS) has been used as a standardized scoring system to quantify abnormal lung aeration in ultrasound images. Lung ultrasound can also assess chronic lung changes over time, the need for surfactant, and guide respiratory care (11–15). Other studies evaluating LUS during the NICU hospitalization have established: (i) higher LUS in infants with BPD compared to infants without BPD, (ii) correlation of LUS with the severity of BPD (mild, moderate, or severe), and (iii) correlation of LUS with increased oxygenation requirements. However, the utility of lung ultrasound beyond the initial NICU hospitalization has not been well established.

To better understand the clinical role of lung ultrasound following NICU discharge, we conducted a cross-sectional study to (1) describe lung ultrasound findings following NICU discharge and (2) assess the association between outpatient LUS abnormalities and respiratory morbidity in preterm children with and without a history of BPD. We additionally examined whether LUS findings varied by BPD severity and corrected age at the time of assessment. We hypothesized that LUS are higher (more abnormal) in children with a history of prematurity and BPD, compared to healthy infants born full term. We also hypothesized that children with severe BPD would have higher LUS compared to children with mild BPD and that older infants with BPD (who are further out from NICU discharge) would have lower LUS compared to younger infants who were recently discharged.

## Material and methods

### Study design & eligibility criteria

Participants were enrolled in a cross-sectional study measuring LUS in children with BPD after discharge from our quaternary level NICU at a university hospital (10, 14, 16). We performed lung ultrasounds in the outpatient clinic setting, weeks to several months after NICU discharge. Our study population included premature children between 0 and 24 months old. As a control population, we recruited healthy children who were born full term without any respiratory conditions. The recruitment and study period took place from August 2022 to April 2023. Each child received only one lung ultrasound assessment during the study period. This study received approval from the UNC IRB (Approval No. 22-0141) and consent was obtained prior to enrollment.

For preterm infants, the inclusion criteria included: (i) birth gestational age of <32 weeks and (ii) the age range of 36 weeks to 24 months (corrected age) at time of ultrasound scan. Infants with major congenital malformations or genetic abnormalities were excluded. For term infants, the inclusion criteria included: (i) birth gestational age of >37 weeks, (ii) age range of 2–24 months at time of ultrasound scan, and (iii) no history of any cardiac or pulmonary conditions (including congenital cardiac defects, pulmonary hypoplasia, pulmonary hypertension, neonatal respiratory distress syndrome, or need for oxygen following birth).

Preterm participants were categorized according to their BPD severity classification, based on the 2019 National Institute of Child Health and Human Development (NICHD) criteria (17), which defines BPD based on the amount of respiratory support needed at 36 weeks post-menstrual age (PMA). This definition defines categories of BPD as: no BPD (the infant did not require supplemental oxygen or respiratory support at 36 weeks PMA); Grade 1 BPD [the infant required supplemental oxygen via nasal cannula <2 liter per minute (LPM) at 36 weeks PMA]; Grade 2 BPD (the infant required ≥2 LPM or noninvasive positive airway pressure at 36 weeks PMA;) and Grade 3 BPD (the infant required invasive mechanical ventilation at 36 weeks PMA). A retrospective review of the electronic health record was completed to ascertain the degree of respiratory support each participant required at 36 weeks PMA.

### Recruitment

**Figure 1:**
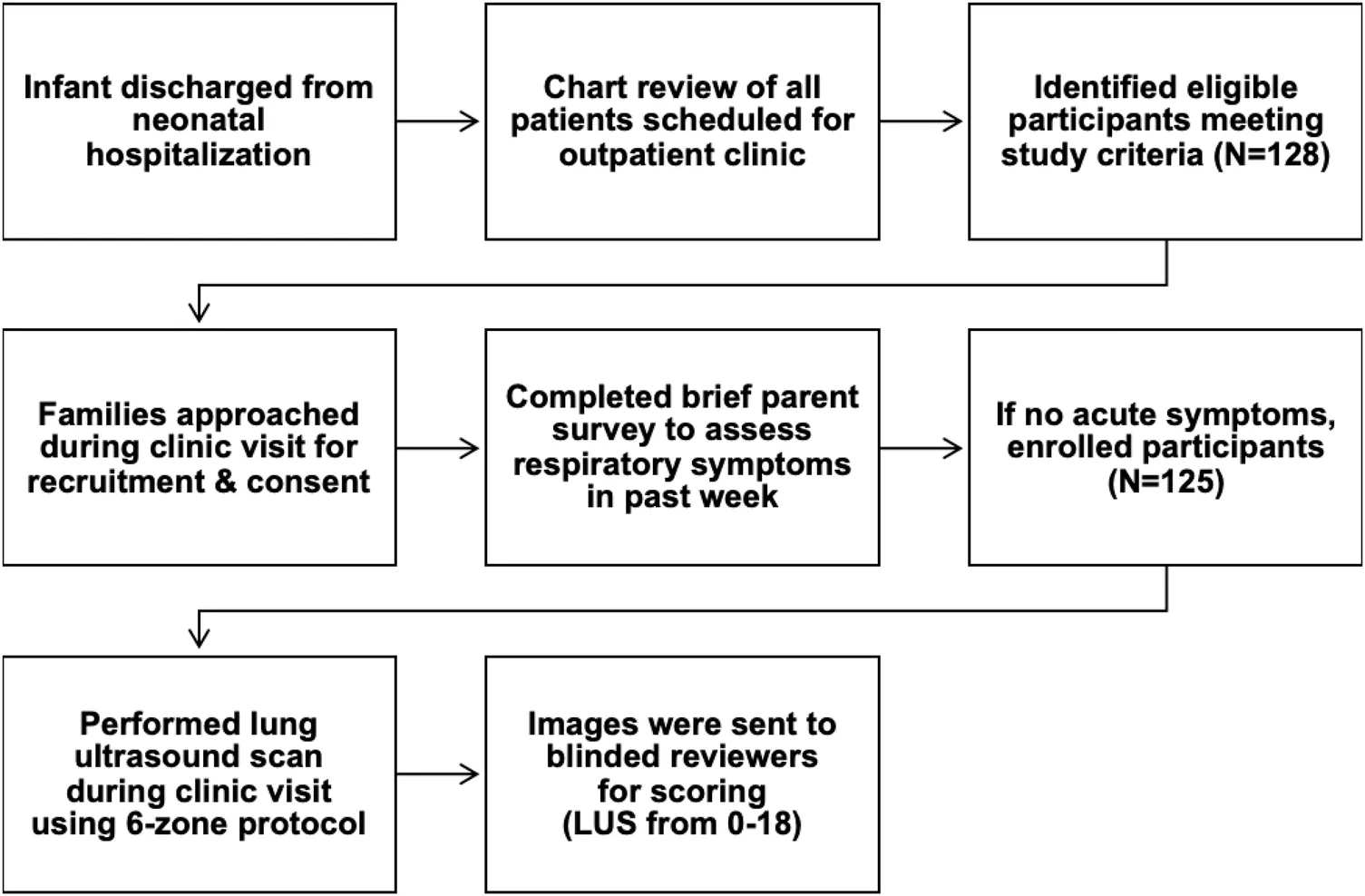
Study flow diagram.

Eligible preterm participants were recruited from two outpatient clinics: NICU developmental follow up clinic and pediatric pulmonology clinic. Eligible term infants were recruited from the general pediatrics clinic. The families were approached for study recruitment during these scheduled outpatient visits. If interested in participating, consent was obtained, and lung ultrasound was performed in the exam room. Families also completed a short 3-question survey about the participant's current respiratory symptoms, recent changes in respiratory support or medications, and respiratory hospital readmissions since NICU discharge. Children with acute respiratory symptoms within the past week were excluded, as we required participants to be at their respiratory baseline, in order to reflect chronic disease patterns rather than acute deterioration. Data pertaining to the neonatal period including patient demographics, respiratory data (duration and level of support of invasive and noninvasive respiratory support, fraction of inspired oxygen), and associated neonatal morbidities were collected for each participant through chart review of electronic health records. Figure 1 describes the flow of participants during the study.

### Lung ultrasound procedure

**Figure 2:**
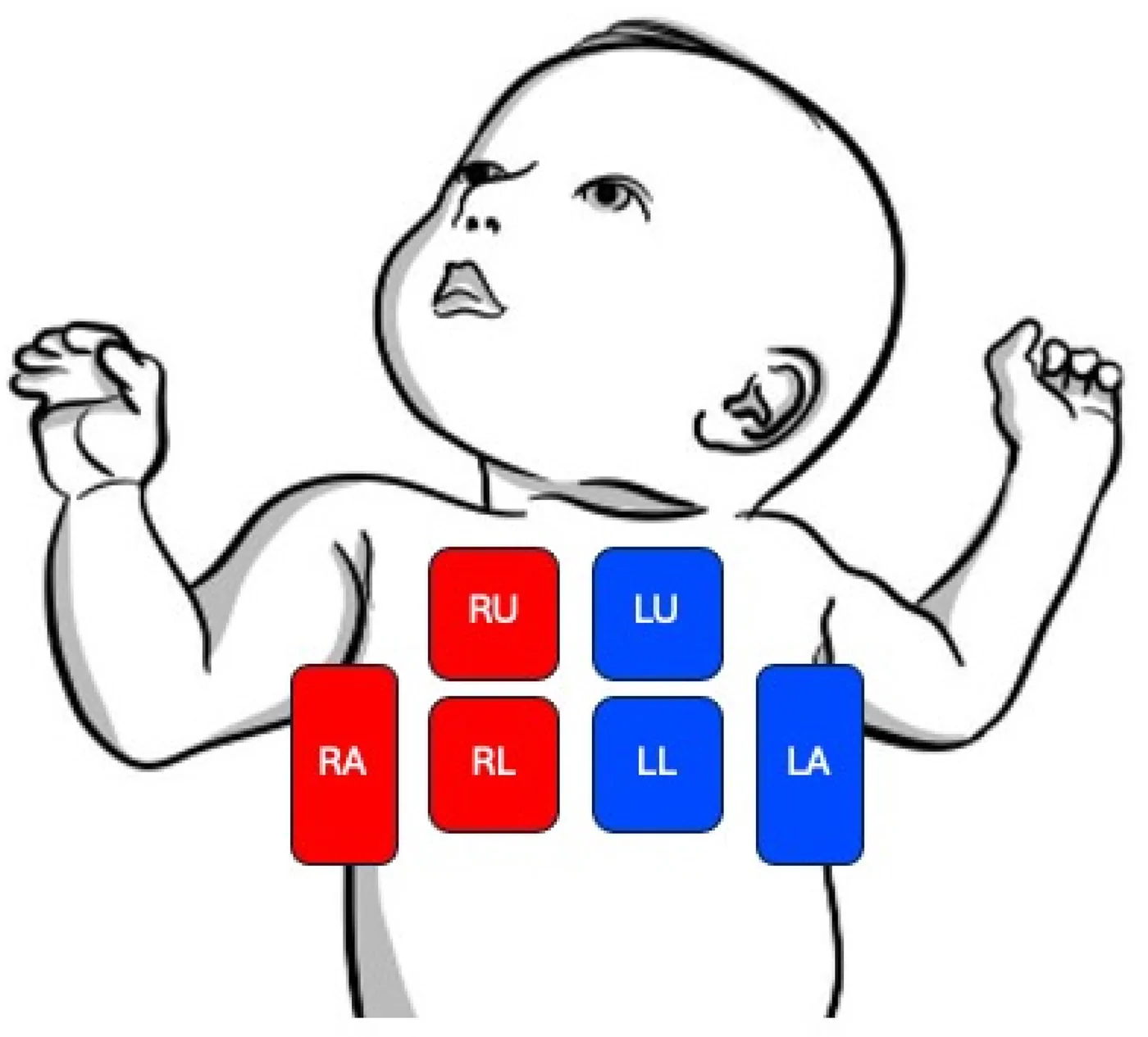
6-zone lung ultrasound protocol. The six zone scanning protocol includes assessment of three lung zones on each lung side. RU, right upper chest; RL, right lower chest; RA, right axillary/lateral chest; LU, left upper chest; LL, left lower chest; LA, left axillary/lateral chest.

**Figure 3:**
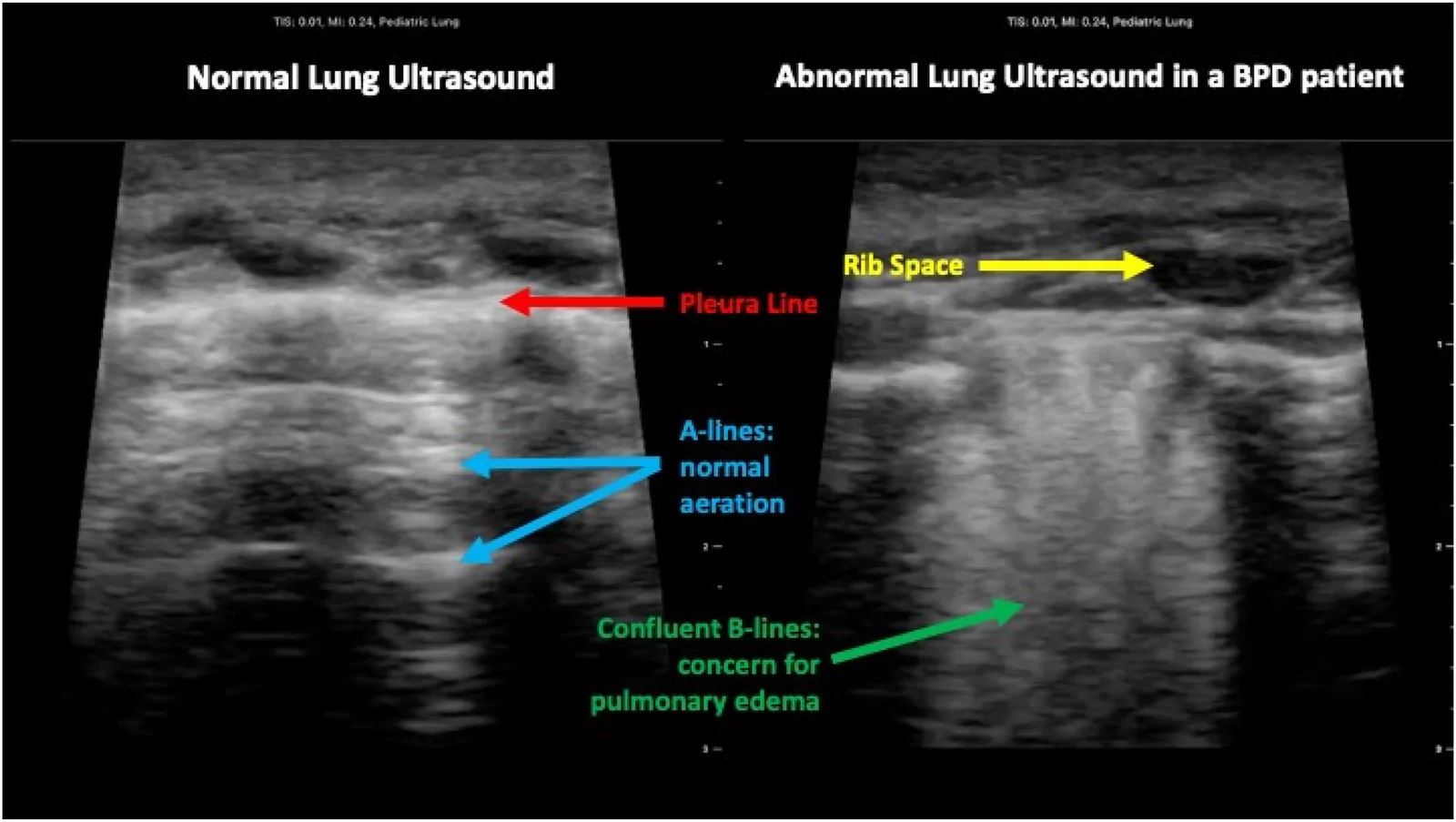
Comparison of normal lung ultrasound to lung ultrasound in BPD.

Lung ultrasound was performed using a handheld ultrasound transducer (Butterfly iQ, Butterfly Network, Inc) and a high frequency “lung” setting on the device. Ultrasounds were performed by the same individual (N.S.) who was trained in ultrasound technique via a formal lung ultrasound practical course. All participants were scanned in the supine position. The scanning protocol included assessment of three lung zones in the right and left lung (upper anterior, lower anterior and axillary/lateral chest) (see Figure 2). Short ultrasound images/clips were obtained in each lung zone and stored in an ultrasound software program for offline analysis by three blinded reviewers (D.P., B.S., R.D.). Each of these reviewers completed a brief lung ultrasound interpretation training course and were provided a scoring rubric with examples. The reviewers assigned a LUS to each image/clip using a scoring system based on recognition of 4 lung patterns: 0 = normal lung with A line pattern and less than three B lines, 1 = interstitial lung changes with more than three nonconfluent B lines, 2 = severe lung interstitial changes with confluent B lines (white lung pattern), and 3 = extensive lung consolidations. The scores from all 6 zones were added for a total LUS between 0 and 18 points. A LUS of 18 indicates the most severe lung aeration abnormalities. A LUS of 0 indicates normal lung aeration. Figure 3 shows a comparison between normal and abnormal lung ultrasound images. Final scores from the three blinded investigators were compared to determine interrater reliability and to assess consistency with scoring (18). These results were de-identified and recorded on a spreadsheet.

### Statistical analysis

Descriptive statistics were used to report the participants' demographic characteristics. The cohort was divided based on the BPD severity subgroups. Chi-squared tests for categorical variables and ANOVA *F*-tests for continuous variables were used to compare the distribution among the subgroups. Inter-observer agreement for total lung ultrasound score (LUS) across the three blinded reviewers was assessed using an intraclass correlation coefficient (ICC) using a two-way random effects model with absolute agreement [ICC (2,1)]. The main outcome was abnormal LUS, which was defined as a LUS greater than 0. Although LUS is an ordinal measure, analyses were selected based on the relationship between the score and disease burden. The association between LUS and BPD was evaluated using a logistic regression model adjusting for age. Logistic regressions were used to assess whether LUS, combined with other important clinical characteristics, had a statistically significant relationship with the likelihood of prior hospital readmission. Area Under the Curve (AUC) of the model was reported to evaluate the relationship accuracy. Significance of all tests was defined as *p* < 0.05. All analyses were performed using SAS software (version 9.4; SAS Institute, Cary NC) or GraphPad Prism (version 10.5, GraphPad Software, Boston, MA).

## Results

128 children met the eligibility criteria and were approached for the study; 125 (97.7%) were consented and enrolled, including 99 preterm infants and 26 healthy term controls. Among preterm infants, 16, 26, and 12 had Grade 1, 2, and 3 BPD, respectively. 45 of the preterm infants did not meet criteria for BPD (Grade 0). Two patients were excluded due to insufficient ultrasound data.

The mean age of the healthy, term participants was 9.2 months, ranging from 2 days old to 24 months old at the time of the lung ultrasound scan. The average age of the preterm participants was 8.5 months (corrected for gestational age), ranging from 41 weeks PMA to 24 months CGA at the time of the ultrasound scan. Infants with BPD were smaller at birth (mean weight 986 grams vs. 1,379 grams) and born at younger gestational ages (26.8 weeks vs. 30 weeks) compared to preterm infants without BPD. Of all preterm infants enrolled, extremely low birth weight (ELBW) was reported in 4%, 37.5%, 65.4%, and 91.7% of infants with Grade 0 (no BPD), Grade 1, Grade 2, and Grade 3 BPD, respectively. The average length of NICU hospitalization was 75.6 days (±26.2), 121.7 days (±44.7), and 273.8 days (±124.4) for infants with Grade 1, Grade 2, and Grade 3 BPD, respectively. Healthy term infants had an average length of newborn hospitalization of 4 days (±7.4). Following discharge, 17 infants with BPD required readmission during the first year of life, compared to 2 infants in the healthy control group. 14 infants with BPD had a history of pulmonary hypertension. 21 children with BPD were being treated with oxygen supplementation and/or tracheostomy/ventilator support at the time of the ultrasound scan. 12 infants with BPD were on diuretic therapy at the time of ultrasound scan. Additional characteristics of the study cohort are listed in Table 1.

**Table 1:** Characteristics of study population (*N* = 125).

	Preterm, no BPD	Grade 1 BPD	Grade 2 BPD	Grade 3 BPD	Healthy Term	*p*-value
	(*N* = 45)	(*N* = 16)	(*N* = 26)	(*N* = 12)	(*N* = 26)	
Gestational age, weeks						<0.0001^b^
Mean (SD)	29.8 (1.61)	29.0 (2.33)	27.0 (2.42)	26.4 (1.96)	39.5 (1.48)	
Range	24.4, 33.3	25.4s, 31.9	22.9, 31.6	24.3, 30.4	36.3, 41.4	
Birth Weight, grams						<0.0001^b^
Mean (SD)	1,378.5 (266.21)	1,282.1 (506.30)	909.5 (342.05)	758.8 (183.30)	3,453.6 (497.55)	
Range	745.0, 2,170.0	590.0, 2,580.0	495.0, 1,766.0	490.0, 1,140.0	2,575.0, 4,546.0	
ELBW, *n* (%)						<0.0001^c^
No	43 (95.6%)	10 (62.5%)	9 (34.6%)	1 (8.3%)	26 (100.0%)	
Yes	2 (4.4%)	6 (37.5%)	17 (65.4%)	11 (91.7%)	0 (0.0%)	
Sex, *n* (%)						0.4524^c^
Female	26 (57.8%)	10 (62.5%)	12 (46.2%)	7 (58.3%)	10 (38.5%)	
Male	19 (42.2%)	6 (37.5%)	14 (53.8%)	5 (41.7%)	16 (61.5%)	
Race, *n* (%)						0.0953^c^
African Am	13 (28.9%)	4 (25.0%)	12 (46.2%)	5 (41.7%)	4 (15.4%)	
Caucasian	19 (42.2%)	7 (43.8%)	6 (23.1%)	7 (58.3%)	13 (50.0%)	
Other	13 (28.9%)	5 (31.3%)	8 (30.8%)	0 (0.0%)	9 (34.6%)	
Antepartum Steroids, *n* (%)						0.0647^c^
No	10 (22.2%)	8 (50.0%)	9 (34.6%)	1 (8.3%)		
Yes	35 (77.8%)	8 (50.0%)	17 (65.4%)	11 (91.7%)		
Surfactant, *n* (%)						0.0009^c^
No	26 (57.8%)	5 (31.3%)	5 (19.2%)	1 (8.3%)		
Yes	19 (42.2%)	11 (68.8%)	21 (80.8%)	11 (91.7%)		
Postnatal Steroids^a^, *n* (%)						<0.0001^c^
No	41 (91.1%)	14 (87.5%)	10 (38.5%)	0 (0.0%)		
Yes	4 (8.9%)	2 (12.5%)	16 (61.5%)	12 (100.0%)		
History of Pulmonary Hypertension, *n* (%)						<0.0001^c^
No	44 (97.8%)	16 (100.0%)	23 (88.5%)	3 (25.0%)	25 (96.2%)	
Yes	1 (2.2%)	0 (0.0%)	3 (11.5%)	9 (75.0%)	1 (3.8%)	
Respiratory Support at Discharge, *n* (%)						<0.0001^c^
LFNC		1 (6.3%)	9 (34.6%)	3 (25.0%)		
Room Air	45 (100.0%)	15 (93.8%)	17 (65.4%)	1 (8.3%)	26 (100.0%)	
Trach/Vent				8 (66.7%)		
History of Hospital Readmission, *n* (%)						
No	41 (91%)	12 (75%)	20 (76.9%)	10 (83.3%)	24 (92%)	
Yes	4 (8.9%)	4 (25%)	6 (23%)	2 (16.7%)	2 (7.6%)	
Length of Stay (days)						<0.0001^b^
Mean (SD)	52.4 (20.10)	75.6 (26.22)	121.7 (44.68)	273.8 (124.39)	4.0 (7.40)	
Range	7.0, 112.0	39.0, 136.0	68.0, 238.0	117.0, 524.0	1.0, 38.0	

^a^ Postnatal steroids refer specifically to 10-day tapered dexamethasone regimen.

^b^ ANOVA *F*-test *p*-value.

^c^ Fisher Exact *p*-value; These measure the *p*-value distribution of each variable across all severity groups.

We performed 750 lung ultrasound scans, which were reviewed independently by three ultrasound experts (D.P., B.S., R.D.). Despite standardized training, substantial variability in scoring was observed across reviewers, resulting in low overall inter-observer agreement (ICC = 0.19, 95% CI: 0.11–0.28). Pairwise agreement between two reviewers was higher; however, heterogeneity in scoring behavior reduced overall reliability. Given very low inter-observer agreement across all three blinded reviewers, primary analyses were conducted using the scores of a single experienced reviewer. This reviewer was selected *post hoc* based on scoring patterns consistent with expected normal findings in healthy controls (i.e., LUS = 0; see Supplementary Materials). Because this selection was not pre-specified using predefined reliability criteria, the findings should be interpreted as exploratory and observer-dependent.

**Figure 4:**
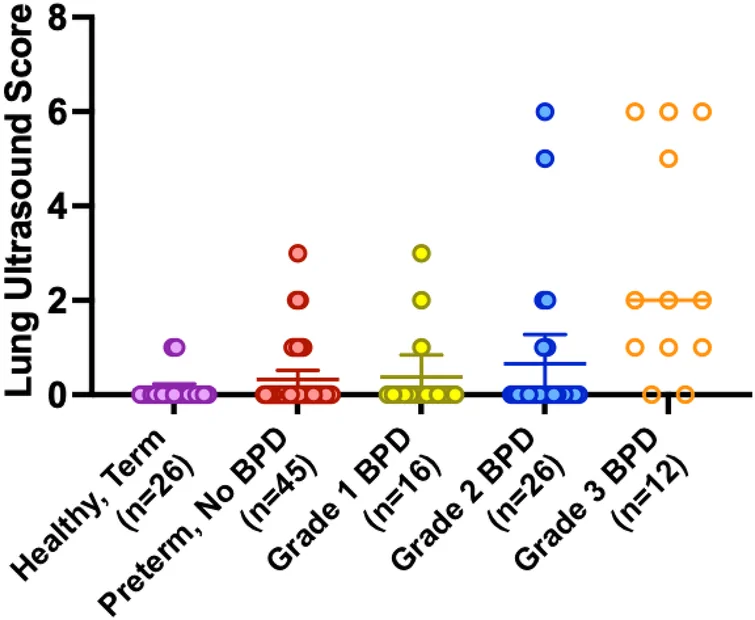
Mean lung ultrasound score (LUS) using BPD severity as Variable. Mean LUS was higher for children with Grade 3 BPD (2.667) when compared to preterm children without BPD or healthy term controls (0.34 vs. 0.11), respectively.

Preterm children with Grade 2 and 3 BPD were significantly more likely to have abnormal lung ultrasound findings compared with healthy term controls. Mean LUS increased for each BPD severity group, with mean LUS of 0.34 for Grade 0, 0.38 for Grade 1, 0.68 for Grade 2, and 2.67 for Grade 3 (Figure 4). Healthy term children had a mean LUS of 0.11. However, the majority of lung ultrasound scans across all groups demonstrated normal lung aeration or LUS of 0 (Table 2).

**Table 2:** Raw comparison of All lung ultrasound scores—contingency table analysis.

LUS BY BPD DEFINITION (FISHER EXACT *P* = 0.0009							
BPD GROUP	Total LUS (outcome)						
	0	1	2	3	5	6	Total
CONTROL	22	3	0	0	0	0	25
0	35	6	3	1	0	0	45
1	13	1	1	1	0	0	16
2	19	2	2	0	1	1	25
3	2	3	3	0	1	3	12
TOTAL	91	15	9	2	2	4	123
FREQUENCY MISSING = 2							

**Figure 5:**
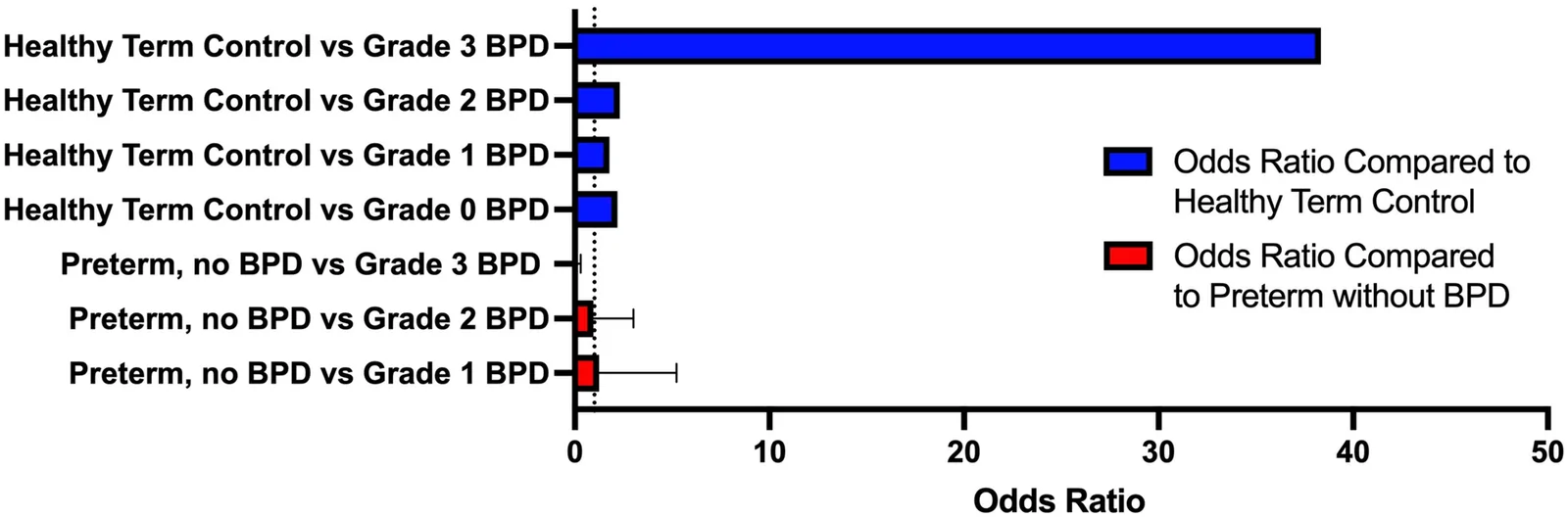
Odds ratio for abnormal LUS in BPD compared to healthy controls. Odds Ratio of abnormal LUS was highest in children with Grade 3 BPD (OR: 38.33, 95% CI: 5.8, 291.3, *p* = 0.0002) when compared to healthy term controls. Odds ratio was lower in children with Grade 0, Grade 1, and Grade 2 BPD, when compared to healthy term controls.

Children with Grade 3 BPD were 38 times more likely to have an abnormal lung ultrasound (OR 38.33; 95% CI: 5.83, 291.25, *p* = 0.0002) compared to healthy control patients (Figure 5). No other statistically significant results were observed among other BPD severity subgroups. We also did not observe any significant associations between LUS and age groups (<12 months or >12 months).

**Figure 6:**
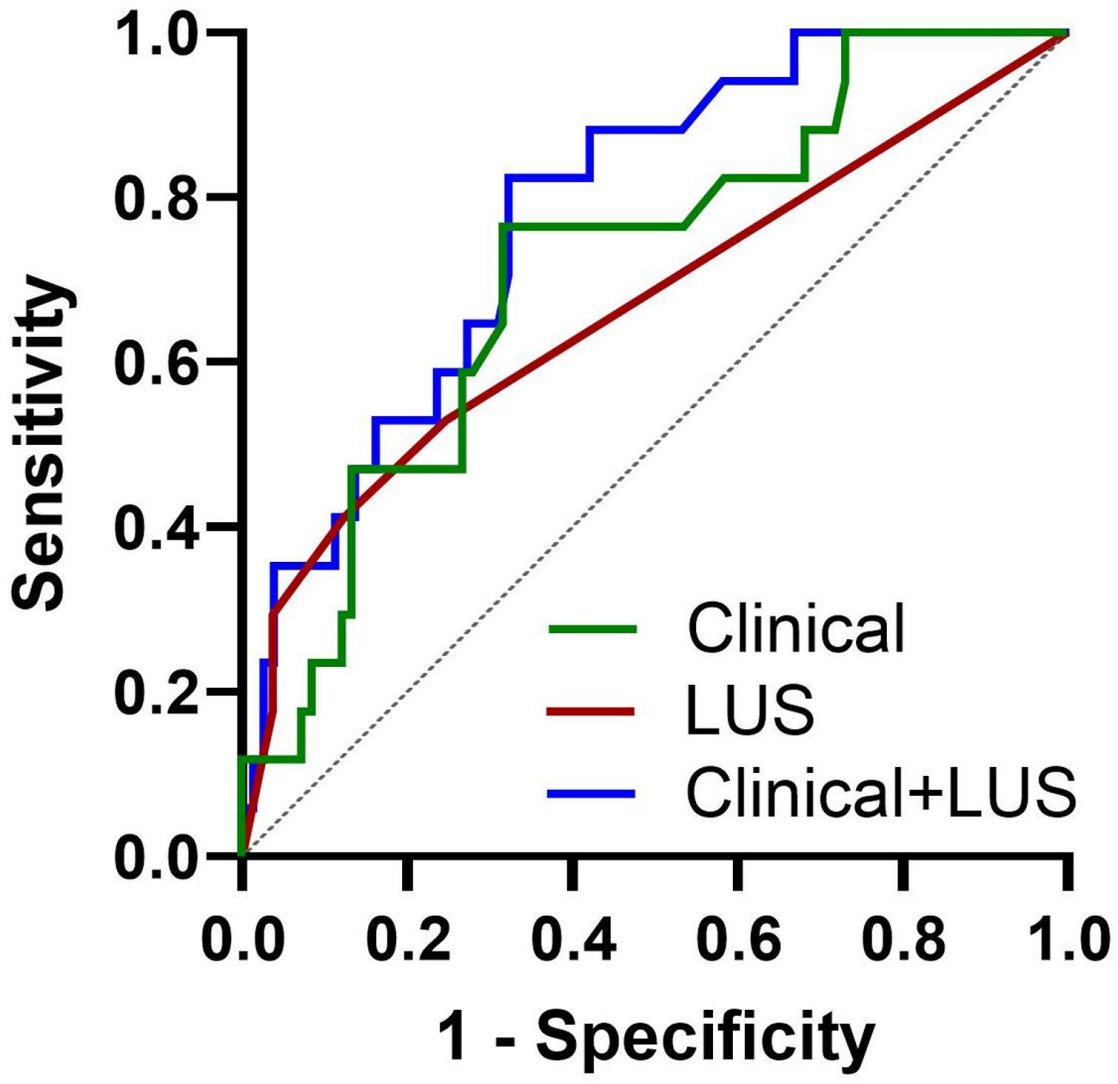
ROC curves model comparisons to describe risk of hospital readmission. Associations between clinical factors, LUS, and hospital readmission among preterm infants. The addition of LUS to a model of clinical characteristics significantly increased the association with hospital readmission, AUC increased from 0.74 to 0.78 (*p* = 0.036 by likelihood ratio test).

Lastly, we assessed the association between abnormal LUS and hospitalization. Of the 99 preterm infants, 17 (13 with BPD) had a history of hospital readmission for respiratory concerns (e.g., bronchiolitis, pneumonia, pulmonary hypertension exacerbations, etc). These readmissions occurred after NICU discharge but prior to the lung ultrasound assessment. Children with acute respiratory symptoms and/or hospital admission within one week of the clinic visit were not eligible to participate in the study. We found that abnormal LUS was 3.5 times more likely in children with a history of hospital readmission for respiratory concerns (*p* = 0.019 by Fisher exact test). To better assess the relationship between LUS and hospital readmissions, we also performed a multivariate model that included multiple known risk factors for hospitalization in children with BPD including gestational age at birth, history of postnatal steroids, respiratory support at discharge (supplemental oxygen and/or mechanical ventilation), and diuretics at discharge. A baseline model including these variables as well as age at lung ultrasound (less than or greater than 12 months) was significantly associated with hospital readmission (AUC 0.74, *p* < 0.002). Adding abnormal LUS (LUS > 0) strengthened the association with hospital readmission (AUC 0.78, *p* < 0.001) with statistically significant improvement (*p* = 0.036 by likelihood ratio test) (Figure 6). Taken together, these findings highlight both the potential utility and important limitations of LUS when applied in the post-discharge setting.

## Discussion

In this cross-sectional study, we provide the first assessment of LUS in a cohort of premature infants beyond the NICU hospitalization, compared with healthy term infants. This study was designed to evaluate associations between outpatient LUS findings and respiratory morbidity, rather than to establish lung ultrasound for routine monitoring. Building on prior observations of LUS during the early stages of BPD in the NICU, we demonstrated a higher likelihood of abnormal LUS among children with severe forms of BPD (Grade 2 and 3) and found that abnormal LUS was often associated with recent respiratory-related hospital readmissions.

Earlier studies described abnormalities in lung aeration patterns measured through lung ultrasound scores in preterm infants with BPD. All but one study investigates lung ultrasound scores only during the NICU hospitalization. High LUS at day 7, 14, and 21 of life was shown to be predictive of a later diagnosis of BPD, compared to lower LUS for infants who did not develop BPD (11). In another study, Adamawa et al. evaluated 27 infants born at <30 weeks of gestation who had lung ultrasound scans between 2 and 8 weeks and reported that infants with BPD had significantly higher LUS than infants who were not diagnosed with BPD (9). In a multicenter study including 240 preterm infants, for whom lung ultrasound was performed at birth and weekly until discharge, lung ultrasound scores throughout the admission were gestational age dependent, significantly correlated with the oxygenation status, and predicted BPD diagnosis at 36 weeks PMA (16). In a similar study of 87 preterm infants, lung ultrasounds were conducted at 28 days, 36 weeks, and at discharge, and showed that LUS correlated with BPD severity and exhibited an improved trend with time toward the point of discharge (19).

In contrast to these NICU-based findings, our post-discharge findings suggest that although some aeration trends persist beyond hospitalization, the majority of children—including many with a history of BPD—demonstrate normal lung aeration patterns (LUS of 0) in the outpatient setting. These findings highlight that while abnormal LUS may identify a subset of higher-risk patients, a normal outpatient LUS does not exclude clinically significant chronic lung disease in children with BPD. This limits the utility of LUS as a standalone monitoring tool in the post-discharge setting. The large proportion of children with normal lung ultrasound scores may reflect partial improvement of lung disease within months following NICU discharge, or alternatively, reduced sensitivity of lung ultrasound for detecting subtle chronic lung changes associated with prematurity when compared to other imaging studies. These findings suggest that a longitudinal approach assessing LUS changes over time may be more informative and is a critical next step in identifying trajectories for normalization of LUS. Finally, since lung structure in BPD may evolve with age, we expected age-related improvement in LUS for older infants. However, we found no statistically significant relationships between various corrected ages and LUS. Scanning children at similar corrected ages may reduce confounders related to age.

Consistent with prior studies (20, 21), we found relatively high rates of hospital readmission (∼24%) within 1 year post NICU discharge in our BPD cohort. Abnormal LUS was associated with hospital readmission, and this association was independent of other clinical risk factors in multivariate analysis. Multiple studies, including Smith et al. and Chye et al, have described higher rates of hospital readmissions (estimated up to 49%–57%) for children born prematurely with BPD, when compared to children without BPD (20, 21). In constructing our clinical models, we selected risk factors commonly used to describe a neonate's severity of lung disease and risk for persisting pulmonary morbidity, including gestational age at birth, history of need for postnatal steroids, respiratory support at NICU discharge, and diuretic therapy at NICU discharge. Adding abnormal LUS (defined as LUS > 0) into the multivariate model significantly improved the model's ability to identify children with a history of re-hospitalization in this population of BPD infants. This information could help guide future randomized case-control studies exploring the use of ultrasound as a non-invasive way to identify children with BPD who are at higher risk of post-NICU cardiopulmonary concerns or complications. In this context, LUS may serve as an adjunctive approach to help identify higher-risk groups that warrant closer clinical observation or consideration of earlier interventions.

Currently, after an infant is discharged from the NICU, clinicians do not regularly use imaging studies for the outpatient management of chronic BPD lung disease, as chest x-ray and chest CT can be time-consuming, expensive, and expose infants to radiation. Therefore, point-of-care lung ultrasound may provide clinicians an opportunity to evaluate long-term lung changes associated with BPD in real-time, during outpatient clinic visits. POCUS also has the potential to advance the field of pediatrics and pediatric pulmonology. Due to the lack of radiation exposure and the low expense, lung ultrasound offers advantages over other imaging studies. Serial lung ultrasound imaging may have potential for tracking lung disease progression or resolution of disease without harm to the patient. In addition, ultrasound imaging may serve as a research endpoint in future studies, although this application requires further validation. Based on information obtained from this cross-sectional study, we are conducting a longitudinal prospective study to follow a cohort of infants with severe BPD from the NICU period through 12 months post-discharge, which we hope will offer further insight into the utility of lung ultrasound for outpatient assessments and may help identify which subgroup of preterm children will benefit most from ultrasound assessments. In parallel studies, we will also compare the diagnostic limitations of LUS in BPD to other imaging studies, such as chest radiograph and chest CT.

## Limitations

Our study has several strengths including enrollment of healthy term controls, detailed comparative analyses incorporating relevant clinical variables, and application of the most recent National Institute of Child Health and Human Development (NICHD) definition of BPD (2019 criteria proposed by Jensen et al.) (3) to evaluate differences across severity groups. The inclusion of outpatient lung ultrasound data beyond the NICU hospitalization addresses an important gap in the current literature.

We also acknowledge several important limitations. First, our study is limited by the relatively small sample size of infants with Grade 3 BPD, which reflects the smaller proportion of surviving infants in this severity category. Larger, multicenter studies will be necessary to improve statistical power and further evaluate this subgroup.

Second, we elected to use a 6-zone ultrasound protocol, as infants spend the majority of their time in a supine position. However, other POCUS studies in older children and adults often employ expanded 8- or 12-zone protocols, which include posterior lung fields and, in some cases, scoring of pleural line abnormalities. The use of a limited scanning protocol may have reduced sensitivity for detecting more subtle or posterior lung pathology.

A key methodological limitation of this study is the low inter-observer reliability demonstrated among blinded reviewers. Formal assessment showed poor overall agreement (ICC = 0.19, 95% CI: 0.11–0.28), reflecting substantial variability in scoring. Although standardized training and scoring rubrics were provided, heterogeneity in scoring behavior was observed across reviewers, and only one reviewer demonstrated scoring patterns consistent with expected normal findings in healthy controls (see Supplementary Materials). As a result, primary analyses were conducted using scores of a single experienced reviewer, which introduces potential observer dependence into the primary analyses and limits generalizability. Because the reviewer was selected *post hoc*, this also introduces potential selection bias and limits the strength of inference regarding clinical applicability. These findings highlight the challenges of implementing lung ultrasound into clinical settings and underscore the need for improved standardization, formal reliability testing, and training benchmarks prior to broader clinical adoption. In future studies, reliability testing should be incorporated early in the study design to ensure consistency and reproducibility of lung ultrasound scoring interpretation.

Additionally, handheld ultrasounds vary widely in sensitivity and image quality. In a recent comparison study of four commonly used handheld ultrasound devices, the Butterfly device was ranked lowest for image quality and lowest for user satisfaction (22). Because the Butterfly iQ was used for data collection in this study, reduced image quality may have further contributed to inter-observer variability.

Finally, lung ultrasound is inherently limited in imaging depth, which may restrict its ability to distinguish airway disease from parenchymal lung disease, or to differentiate BPD from other forms of chronic lung disease in infancy, particularly when compared to chest CT or MRI. However, these other imaging modalities carry increased risks related to radiation exposure or the need for sedation. Continued investigation of ultrasound-based approaches remains warranted. Our future research will build upon these findings and incorporate strategies to address these limitations.

Given these limitations, this study should be interpreted as hypothesis-generating and descriptive of associations observed by a single experienced reviewer, rather than as validation of lung ultrasound as a reproducible clinical monitoring tool. Prospective studies with predefined reliability criteria and standardized interpretation are needed before clinical implementation.

## Conclusion

Lung POCUS may serve as a valuable adjunctive imaging tool for outpatient assessment of respiratory disease in children with severe BPD; however, normal findings are common and do not exclude clinically significant chronic lung disease. Further prospective studies are needed to define its optimal role and limitations in post-discharge care.

## Data Availability

The raw data supporting the conclusions of this article will be made available by the authors, without undue reservation.
