# Correction: Outpatient assessment of bronchopulmonary dysplasia using point of care lung ultrasound

**DOI:** 10.3389/fped.2026.2018239

**Published:** 2026-10-22

**Authors:** Nicole Stephenson, William Stoudemire, Daniel Park, Lang Li, Charles Esther, Ria Dancel, Benjamin Smith, Feng-Chang Lin, Stephanie D. Davis

**Affiliations:** 1 Department of Pediatrics, The University of North Carolina School of Medicine, Chapel Hill, NC, United States; 2 Department of Biostatistics, Gillings School of Global Public Health, The University of North Carolina at Chapel Hill, Chapel Hill, NC, United States; 3 Department of Medicine, The University of North Carolina School of Medicine, Chapel Hill, NC, United States; 4 Department of Radiology, The University of North Carolina School of Medicine, Chapel Hill, NC, United States

**Keywords:** bronchopulmonary dysplasia, lung point-of-care ultrasound, lung ultrasound, lung ultrasound scores, respiratory morbidity

The funder “American Pediatric Association's 2024 Research in Academic Pediatrics Investigator Development (RAPID) Scholar” was erroneously omitted from the Funding statement.

The original version of this article has been updated.

