# Supplementary material for: Outpatient assessment of bronchopulmonary dysplasia using point of care lung ultrasound
Source: Front Pediatr. 2026 Apr 2;14:1733693. doi: 10.3389/fped.2026.1733693 (PMC13085926; doi:10.3389/fped.2026.1733693)
Supplement: Supplementary file 1 [file Supplementaryfile1.docx]

**SUPPLEMENTAL MATERIALS**

**Justification for Using Reviewer 1 Only for Data Analysis**

**Figure 1** shows LUS for all healthy controls, along with results of a one-sample T-test on whether the findings were different from zero. Reviewer 1 reliably scored healthy controls as zero (which would be expected in healthy lung tissue). Reviewers 2 and 3 scored healthy controls as significantly higher than zero.

**FIGURE 1:
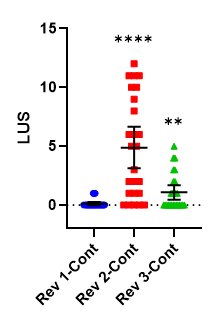
**

**Figure 2** shows LUS for control and BPD populations for all reviewers along with Mann-Whitney tests for differences in scores between control and BPD. Only Reviewer 1 had scores that were statistically different between control and BPD groups. The data shows objectively that LUSs by Reviewers 2 and 3 did not distinguish ultrasound findings associated with BPD.

**FIGURE 2:
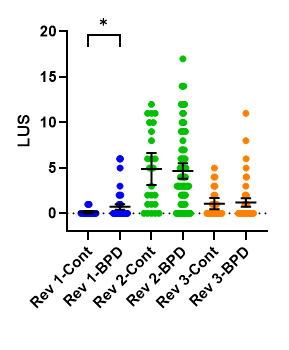
**
